# A nemertean excitatory peptide/CCHamide regulates ciliary swimming in the larvae of *Lineus longissimus*

**DOI:** 10.1186/s12983-019-0326-9

**Published:** 2019-07-10

**Authors:** Daniel Thiel, Philipp Bauknecht, Gáspár Jékely, Andreas Hejnol

**Affiliations:** 1 Sars International Centre for Marine Molecular Biology, University of Bergen, Thormøhlensgate 55, 5006 Bergen, Norway; 2 Max Planck Institute for Developmental Biology, Spemannstraße 35, 72076 Tübingen, Germany; 3 Living Systems Institute, University of Exeter, Stocker Road, Exeter, EX4 4QD UK

**Keywords:** Neuropeptide, GPCR, CCHamide, Excitatory peptide, Nemertea, GGNG, Locomotion, Ciliary beating

## Abstract

**Background:**

The trochozoan excitatory peptide (EP) and its ortholog, the arthropod CCHamide, are neuropeptides that are only investigated in very few animal species. Previous studies on different trochozoan species focused on their physiological effect in adult specimens, demonstrating a myo-excitatory effect, often on tissues of the digestive system. The function of EP in the planktonic larvae of trochozoans has not yet been studied.

**Results:**

We surveyed transcriptomes from species of various spiralian (Orthonectida, Nemertea, Brachiopoda, Entoprocta, Rotifera) and ecdysozoan taxa (Tardigrada, Onychophora, Priapulida, Loricifera, Nematomorpha) to investigate the evolution of EPs/CCHamides in protostomes. We found that the EPs of several pilidiophoran nemerteans show a characteristic difference in their C-terminus. Deorphanization of a pilidiophoran EP receptor showed, that the two splice variants of the nemertean *Lineus longissimus* EP activate a single receptor. We investigated the expression of EP in *L. longissimus* larvae and juveniles with customized antibodies and found that EP positive nerves in larvae project from the apical organ to the ciliary band and that EP is expressed more broadly in juveniles in the neuropil and the prominent longitudinal nerve cords. While exposing juvenile *L. longissimus* specimens to synthetic excitatory peptides did not show any obvious effect, exposure of larvae to either of the two EPs increased the beat frequency of their locomotory cilia and shifted their vertical swimming distribution in a water column upwards.

**Conclusion:**

Our results show that EP/CCHamide peptides are broadly conserved in protostomes. We show that the EP increases the ciliary beat frequency of *L. longissimus* larvae, which shifts their vertical distribution in a water column upwards. Endogenous EP may be released at the ciliary band from the projections of apical organ EP positive neurons to regulate ciliary beating. This locomotory function of EP in *L. longissimus* larvae stands in contrast to the repeated association of EP/CCHamides with its myo-excitatory effect in adult trochozoans and the general association with the digestive system in many protostomes.

**Electronic supplementary material:**

The online version of this article (10.1186/s12983-019-0326-9) contains supplementary material, which is available to authorized users.

## Introduction

Neuropeptides are neurotransmitters that are involved in the regulation of most behavioral and physiological processes in animals. Many neuropeptide systems are ancestral to bilaterians and orthologous neuropeptides are deployed in the different bilaterian lineages independent of their nervous system organization [1–3]. Only few of the orthologous neuropeptides are well conserved in their amino acid sequences between the different bilaterian lineages and it is often the orthology of their receptors, that reveals their homology [1–4]. For example, the protostome orthologs of vertebrate neuromedin -B/bombesin- and endothelin-related neuropeptides, the CCHamide and excitatory peptide (EP), are neuropeptides where the orthology of the deuterostome and protostome peptidergic systems could only be detected because of their receptor similarity [2, 3, 5].

**Fig. 1:**
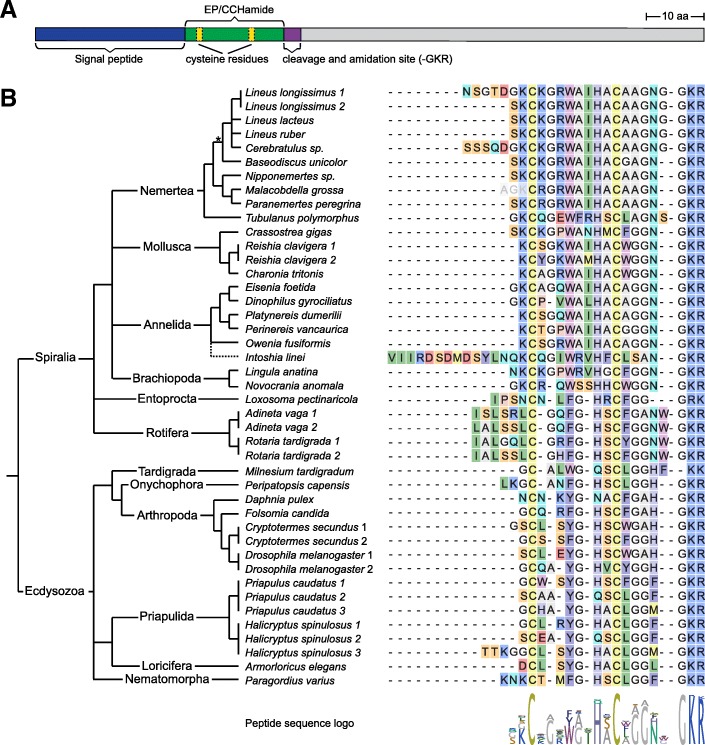
Protostome EP/CCHamide sequences. **a** Schematic structure of the 116 amino acid long *L. longissimus* EP2 precursor. Scale bar on the upper right indicates the length of 10 amino acids. **b** Alignment of the predicted EP/CCHamide peptides of different protostomes with the phylogenetic relationship of the different taxa. C-terminal GKR, GKK or GRK residues indicate the precursor cleavage site and a C-terminal amidation of the residue N-terminal to the glycine, a missing glycine residue (e.g. *M. tardigradum*) indicates only cleavage without amidation. Peptide sequence logo was created from the alignment. Phylogeny is depicted according to Dunn et al. 2014 [30], annelid phylogeny according to Struck et al. 2015 [31] with Orthonectida as an annelid taxon [32], arthropod phylogeny according to Yeates et al. 2016 [33] and nemertean phylogeny according to Andrade et al. 2014 [34] and Kvist et al. 2015 [35]. Dashed line indicates unclear relationship, asterisks indicate the heteronemertean branch. Full precursor sequences are available in Additional file 1

The EP was initially discovered in the earthworms *Eisenia foetida* and *Pheretima vittata* [6] and has since been identified in many annelid and mollusk species. It has been described under various names depending on the taxon and due to either its myo-excitatory effect or its C-terminal structure (see Table 1 for species, peptide names and references). The arthropod ortholog CCHamide was first discovered in the silk worm *Bombyx mori* [23] and is known from various arthropods, including insects [3, 23, 24], crustaceans [3, 24–26], myriapods [27] and chelicerates [24, 28, 29], with its nomenclature based on the presence of two conserved cysteine residues and an amidated C-terminal histidine residue. Because of the presence of the two cysteine residues, the usually amidated C-terminus and a similar precursor structure in CCHamides and EPs (Fig. 1a), the two peptides were already recognized as possible orthologs [2, 3, 17] before the corresponding receptors were known. This orthology hypothesis was then confirmed with the deorphanization of their orthologous receptors [12, 24]. Notably, the CCHamide system duplicated within the insect lineage into two distinctive CCHamides with two distinctive CCHamide receptors, where each of the two peptides seems to specifically activate its own, corresponding receptor paralog [5, 24]. Experiments showed that CCHamide is involved in the regulation of feeding, sensory perception and the control of insulin like peptides in *Drosophila melanogaster* [36–38] and connected to feeding in other insects [5, 39]. Its expression in different larval or adult insects is connected to the digestive system [23, 38–42]. Experiments with EP showed a myo-excitatory effect that included digestive tissues of oligochaetes [6], leeches [7, 8], and a gastropod species [15], and association with digestive tissues was also shown by immunohistochemistry on a polychaete [10]. (See also Table 2 for functional and anatomical association of EP/CCHamides). The myo-excitatory effect and the expression of EP was observed on tissues of adult animals.

**Table 1:** Discovery and nomenclature of trochozoan EPs

Clade	Species	Peptide name	Reference
Annelida	*Eisenia foetida, Pheretima vittata*	GGNG peptide	[6]
	*Hirudo nipponia, Whitmania pigra*	GGNG peptide, LEP (leech excitatory peptide), EEP (earthworm excitatory peptide)	[7, 8]
	*Eisenia foetida*	GGNG peptide, LEP, EEP,	[9]
	*Perinereis vancaurica*	GGNG peptide, PEP (polychaete excitatory peptide)	[10]
	*Capitella teleta* *(Hirudo japonica, Hirudo medicinalis, Alvinella pompejana)*	GGNG	[11]
	–	EP (excitatory peptide)	[2]
	*Platynereis dumerilii*	EP (excitatory peptide)	[12, 13]
	*Dinophilus gyrociliatus, D. taeniatus, Trilobodrilus axi*	EP	[14]
Mollusca	*Thais clavigera*	GGNG peptide, TEP (*Thais* excitatory peptide)	[15, 16]
	*Lottia gigantea*	GGNG	[17]
	*Crassostrea gigas*	GGNamide	[18]
	*Theba pisana*	GGNG	[19]
	*Sepia officinalis*	GGNG, GGNamide	[20]
	*Charonia tritonis*	GGNG	[21]
	*Patinopecten yessoensis*	GGNamide	[22]

**Table 2:** Association of EP/CCHamide peptidergic signaling based on expression, peptide detection and functional analysis of previous studies

Clade	Species	Results with regard to EP/CCHamide	Reference
Annelida	*Eisenia foetida, Pheretima vittata*	Isolation of EP from gut tissue as well as whole bodies and excitation of gut tissue by EP application.	[6]
	*Hirudo nipponia,*	Excitation of the crop gizzard by EP application.	[7]
	*Eisenia foetida*	EP binding capacity is high in anterior part of digestive tract and the nephridia.	[9]
	*Whitmania pigra*	EP immunoreactivity in supra-esophageal ganglion, circum-esophageal connective, sex segmental ganglion.	[8]
	*Perinereis vancaurica*	EP immunoreactivity in CNS, epithelial cells of pharynx and epidermal cells.	[10]
Mollusca	*Thais clavigera*	Excitation of esophagus and penial complex by EP application, EP immunoreactivity in CNS and nerve endings of the penial complex.	[15]
	*Thais clavigera*	EP1 expression in sub-esophageal, pleural, pedal and visceral ganglion and EP2 expression in pedal and visceral ganglion.	[16]
Hexapoda	*Bombyx mori* - larvae	CCHa expression in the central nervous system and the midgut.	[23]
	*Phormia regina* - adults	CCHa2 injection stimulates feeding motivation (measured by the proboscis extension reflex at different sugar concentrations).	[5]
	*Delia radicum* - larvae	CCHa1 was exclusively detected in the gut.	[41]
	*Spodoptera exigua* - larvae	CCHa1 and CCHa2 are expressed in the larval gut and brain. Starvation increased CCHa1 expression in larvae.	[39]
	*Drosohila melanogaster* - larvae & adults	High CCHa2 expression in gut and low expression in brain; high CCHa2 receptor expression in brain and low expression in gut.	[40]
	*D. melanogaster* - adults	Upregulation of CCHa (1?) in the brain of starved animals. RNAi knockdown of the CCHa1 receptor and CCHa1 receptor mutants showed an abolishment of a starvation-induced increase in olfactory responsiveness.	[36]
	*D. melanogaster* - larvae & adults	Distinct CCHa1 and CCHa2 immunoreactivity in the digestive tract in both larvae and adults.	[42]
	*D. melanogaster* - larvae	CCHa2 is highly expressed in fat body and slightly in gut, CCHa2 receptor is expressed in few endocrine cells in the brain including insulin like peptide (ILP) 2 producing cells. Starvation reduces CCHa2 expression. CCHa2 receptor mutants showed no change in ILP 2 and 3 expression but reduced ILP 5 expression. CCHa2 mutants show growth retardation and developmental delay. CCHa2 mutants show reduced ILP 5 expression and reduced body weight.	[38]
	*D. melanogaster* - larvae & adults	Larvae: CCHa2 mutants show reduced feeding rate/activity and have a delayed development. Larvae and Pupae have reduced expression of insulin like peptide 2 and 3. CCHa2 is highly expressed in gut and slightly in brain. No effect detected for CCHa1. Adults: CCHa2 mutants show reduced feeding and reduced locomotory activity. No effect detected for CCHa1.	[37]
Crustacea	*Marsupenaeus japonicus –* juvenile/adults	Highest expression of CCHa in ventral nerve cord, brain, eyestalks and gills, only low expression in intestines and stomach tissue. No effect of starvation on CCHa expression.	[43]
	*Nephrops norvegicus* - adults	Tissue specific transcriptome detection of two CCHa’s in brain, thoracic ganglia and eyestalks, but not in hepatopancreas or ovaries.	[26]

Annelids and molluscs, as well as other closely related taxa such as brachiopods and nemerteans, also possess planktonic larvae that usually metamorphose into morphologically different, benthic adults. This ciliated larva is the name giving characteristic of the spiralian taxon Trochozoa [30, 44–47]. While antibodies against neuropeptides (usually FMRFamide) have been used widely to describe the nervous system of trochozoan larvae [48–56], there are comparably few studies that investigated the behavioral effect of neuropeptides in such larvae [57–61] and neither behavioral nor immunohistochemical studies investigated the EP/CCHamide in trochozoan larvae. In nemerteans no functional studies with neuropeptides have been reported so far. One group of nemerteans, the pilidiophorans [34, 35, 62], have a planktotrophic and long living pilidium larva (with a few exceptions [63, 64]). Within such a pilidium larva, the juvenile worm develops from initially isolated imaginal discs that eventually fuse and overgrow the larval gut until it hatches after several weeks and often devours its own larval tissue [64, 65]. The larval nervous system lacks a typical neuropil and comprises an apical organ that consists of an apical plate with densely arranged secretory cells at the base of a prominent apical tuft, and neurons that are associated with the digestive system and a nerve net that loosely covers the body and eventually innervate a prominent nerve underneath the ciliary band [51, 65, 66].

Here we report on the evolution of EP/CCHamide orthologs in ecdysozoans and spiralians with a focus on nemerteans. We test the activation of a single EP receptor by two EP splice variants in the nemertean *Lineus longissimus*, the EP expression in the pilidium larvae and juveniles of *L. longissimus*, and the influence of the EP on the behavior of *L. longissimus* larvae.

## Material and methods

### Bioinformatics

Publicly available transcriptomic or genomic sequencing data were scanned for EP precursors or EP receptors. *Adineta vaga* (Ensembl: AMS_PRJEB1171), *Armorloricus elegans* (NCBI: SRR2131253), *Baseodiscus unicolor* (NCBI: SRR1505175), *Cerebratulus* spec. (NCBI: SRR1797867), *Dinophilus gyrocilatus* (NCBI: SRR4039542), *Halicryptus spinulosus* (NCBI: SRR2682062), *Intoshia linei* (NCBI: PRJNA316116), *Lineus lacteus* ([67] dryad data package), *Lineus longissimus* (NCBI: SRS1118155), *Lineus ruber* ([67] dryad data package), *Loxosoma pectinaricola* ([67] dryad data package), *Malacobdella grossa* ([67] dryad data package), *Milnesium tardigradum* (NCBI: SRR1598869), *Nipponemertes* spec. (NCBI: SRR1508368), *Novocrania anomala* (NCBI: SRS1118148), *Owenia fusiformis* (NCBI: SRS590031), *Paragordius varius* (NCBI: PRJEB19315), *Paranemertes peregrine* ([67] dryad data package), *Peripatopsis capensis* (NCBI: SRR1145776), *Priapulus caudatus* (NCBI: SRR1212719), *Rotaria tardigrade* (NCBI: SRR2430032), *Tubulanus polymorphus* ([67] dryad data package). Raw reads from NCBI were assembled with a pipeline that used Trimmomatic for read preprosessing [68], SPAdes for error correction [69] and Trinity for the assembly [70]. Assembled transcriptomes and those from the [67] dryad data package were translated into protein sequences with TransDecoder (https://github.com/TransDecoder/TransDecoder) (Additional file 9, Additional file 10, Additional file 11).

Publicly available sequences from NCBI and from publications that are listed in Table 1 and Table 2 were used as BLAST reference sequences. Neuropeptide precursor sequences were tested for the presence of a signal peptide using SignalP 4.1 [71]) and Signal-3 L 2.0 [72] and cleavage sites were predicted with the NeuroPred online application (http://stagbeetle.animal.uiuc.edu). Receptor sequences were aligned with ClustalX v2.1 [73], non-conserved regions where removed with TrimAl (using the gappyout option) [74], the phylogenetic tree was calculated with FastTree v2.1 [75] (using the LG amino acid substitution model) and visualized with FigTree v1.4.3 (https://github.com/rambaut/figtree/releases).

### Receptor activation assay

For the receptor activation assay the protocol of [12] was followed. The full ORF of the *L. longissimus* EP-receptor sequence was amplified by PCR from larval cDNA and was cloned into the mammalian expression vector pcDNA3.1(+). The vector was transfected into CHO-K1 cells together with a promiscuous G_α_-16 protein encoding plasmid and a calcium sensitive luminescent apoaequorin-GFP fusion protein encoding plasmid (G5A). Synthetic peptides of the predicted, mature *L. longissimus* EP1 and EP2 (customized from GenScript; sequences as described below) were diluted to different concentrations, the dose-dependent luminescence response was measured in a plate reader (BioTek Synergy Mx and Synergy H4, BioTek, Winooski, USA) and analyzed with Prism 6 (GraphPad, La Jolla, USA).

### Animal rearing

Adult *Lineus longissimus* (Gunnerus, 1770) were collected at different collection sites close to the Marine Biological Station Espegrend (University of Bergen) in Norway. *L. longissimus* were kept together at 12 °C in natural seawater with an approximate salinity of 33–35 ppt, until spawning occurred naturally during Spring. Larvae were kept in 5-l beakers with slow rotators and were fed *Rhinomonas* sp. algae. Juveniles were retrieved by naturally occurring metamorphosis after about 4–6 weeks of continues rearing.

### Immunohistochemistry

Antibodies against the C-terminal CAAGNGamide sequence of the *Lineus* excitatory peptide were raised in rabbits (genscript) and the antibodies were purified from the blood serum with a Sulfolink™ Immobilization Kit (ThermoFisher Scientific) according to [48]. *L. longissimus* larvae and juveniles were relaxed in an 8% MgCl_2_ x 6H_2_O solution in distilled water for 10 min, fixed in a 4% Formaldehyde solution in seawater for 1 h, washed 5 × 5 min in PBS + 0.1% Tween (PTw), transferred into methanol and stored at − 20 °C. After rehydration in PTw, specimens were incubated in PBS + 0.5% Triton X for 4 h at room temperature, transferred into a 0.1 M Tris pH 8.5 + 0.1% Tween solution (THT), blocked for 1 h in a 5% normal goat serum (NGS) solution in THT and incubated with the primary antibody in THT + 5% NGS for 3 days (approximately 1 ng/μl anti-EP antibody from rabbit and 0.5 ng/μl anti-tyrosinated tubulin antibody from mouse). The primary antibodies were washed 2 × 5 min in THT containing 0.9 M NaCl, washed 5 × 15 min in PBS + 0.2% Triton X + 0.1% BSA (PBT), then washed 5 × 30 min in PTw, transferred into THT, blocked for 1 h in THT + 5% NGS and incubated with the secondary antibodies in THT + 5% NGS overnight (Alexa 488 anti-rabbit and Alexa 647 anti-mouse). Specimens were then washed 5 × 15 min in PBT, 2 × 30 min in PTw, incubated 1 h in PTw containing 1 μg/ml DAPI, washed 2 × 30 min in PTw and transferred into 70% glycerol before mounting in 70% glycerol. As a control to test the antibody specificity, antibodies were preabsorbed in a 2 mM solution of synthetic full-length EP in THT + 5% NGS during the blocking step of the primary antibody.

### Vertical swimming assay

Two to three week old larvae of *L. longissimus* were recorded in transparent columns (6.5 cm height / 3.7 cm length / 2.2 cm width) to compare the vertical distribution of EP-exposed and untreated larvae. For each treatment and replicate more than 200 larvae per column were recorded with a “DMK31AU03” camera (The Imaging Source®) with LED illumination in a darkened box to avoid uncontrolled effects due to the possibility of photo-behavior of the larvae. All treatments and recordings were repeated twice, each time with treatment and control being recorded next to each other. The videos were processed and analyzed with “Fiji” [76] and the repeats were averaged afterwards. (Fiji measurements: 1. Background elimination using Z-project (Average Intensity of all frames) and Image calculator (substract Z-projection from all frames of the video), 2. Conversion of moving larvae to black dots using individual threshold parameter, 3. Combining distribution of larvae over time intervals of 5–10 s using Z-project (Standard Deviation of the corresponding frame sequences), 4. Measurement of larval distribution by dividing the height of the column into 50 squares and using the ‘Measure’ function on each height division, 5. Comparing the measurements in an table to get a horizontal profile of distribution of larvae.) The distributions were compared and tested with a two sample Kolmogorov-Smirnov test (ks.test in “R”).

### Measurement of the ciliary beat frequency of *L. longissimus* larvae

First attempts to immobilize *L. longissimus* larvae between a microscopic glass slide and cover slip failed, as the larvae were either slowly moving away or they got stuck and the cilia that touched the glass stopped beating rhythmically. Therefore, pulled glass holding-capillaries with an opening of about 50–70 μm were used to immobilize the pilidium larvae, similar to [77]. Larvae were caught with the capillaries at their apical tip between a glass slide and a cover slip that was completely filled with seawater. Reagents were added on one side of the cover slip with a pipette and simultaneously soaked off from the other side with a tissue paper, enabling paired measurements of the ciliary beating from individual larvae, under control conditions, after soaking in peptides and after peptide washout in. The ciliary beating was recorded with a DMK23UV024 camera (The Imaging Source) with 50 frames per second. We used 2–3 week old larvae as this size enabled us to use a holding pipette that has a strong enough suction to keep the larvae from getting washed away when we changed the solution.

Preliminary measurements were taken to determine the larval excitation that was triggered by being immobilized and sucked to a holding capillary or by the water flow from changing the solutions. The tests showed that the ciliary beating slowly decreased after larvae were caught with the holding capillary, reaching after about 10 mins a point where the beat frequency did not further decrease. The next test showed that an initial increase in the ciliary beat frequency caused by an exchange of the solution decreased back to the normal frequency in less than 2 min. Test with several exchanges in frequent intervals showed that the ciliary beating was always back to the initial frequency after less than 2 min. Based on these preliminary tests, measurements were taken as follow: Larvae were caught with a glass capillary, the slide got transferred to the microscope and after about 10 min the seawater was exchanged with fresh seawater twice in a row with a waiting time of 2 min after each exchange, before the control condition was recorded. The seawater solution was then exchanged a third time with seawater containing one of the peptides, and after 2 min the ciliary beating under the influence of EP was recorded. Afterwards the larvae were washed for 15 min with several exchanges of fresh seawater before the ciliary beating for the washout was recorded.

Generally, the beating of apical and lateral ciliary bands was tested for 12 larvae under the influence of EP1 and 12 larvae under the influence of EP2, measuring control, peptide exposed and washout conditions for every larva. The videos were processed with Fiji (“plot Z-axis Profile” function to measure the frequency), and differences in the ciliary beat frequency were tested for significance with a paired t-test.

## Results

### Transcriptome analyses show a wide distribution of EP/CCHamide orthologs in protostomes and an extended C-terminal region in several heteronemerteans

We identified spiralian orthologs of EP/CCHamide in transcriptomes of nemertean, brachiopod, entoproct and rotifer species, and ecdysozoan orthologs in tardigrade, onychophoran, priapulid, loriciferan and nematomorph species, showing the wide distribution of EP/CCHamide within protostomes (Fig. 1b, Additional file 1). Nearly all peptides possess two conserved cysteine residues, a proven or predicted amidated C-terminus (Fig. 1b) and a propeptide structure with a single copy of the bioactive peptide directly located after the signal peptide (Fig. 1a, Additional file 1). The main exception seems to be the EP ortholog in the tardigrade *Milnesium tardigradum,* which lacks a C-terminal glycine as a substrate for amidation (Fig. 1b) and also has a precursor where the signal peptide and the EP/CCHamide peptide are separated by several amino acids (Additional file 1) – although a similar precursor structure has also been described for the leech *Hirudo nipponia* [78]. The NeuroPred cleavage prediction of the nemertean *M. grossa* EP precursors also predicted an additional peptide between the signal peptide and the mature EP, which would lead to a cleavage at the lysine residue N-terminal of the first cysteine (Fig. 1b, Additional file 1), however, mass spectrometric evidence from other spiralians did not show any cleavage at this position [6, 7, 10, 15]. The name-giving C-terminal histidine residue of the arthropod CCHamide is only present in Arthropoda and Onychophora. This residue seems more variable within ecdysozoans, with methionine, phenylalanine or leucine residues in priapulid, nematomorph and loriciferan species. All investigated spiralians share an asparagine residue in this position, although while in most taxa this seems to be the most C-terminal residue, both investigated rotifer species and several nemerteans possess an extended C-terminal region with an additional amino acid. The closely related heteronemertean *Cerebratulus* and *Lineus* species all possess an additional glycine residue. This glycine residue seems to have evolved within the heteronemertean lineage, as neither the more distantly related heteronemertean *Baseodiscus unicolor*, nor the non-heteronemerteans showed this additional residue.

### Two *L. longissimus* excitatory peptide isoforms activate a single *L. longissimus* EP receptor

**Fig. 2:**
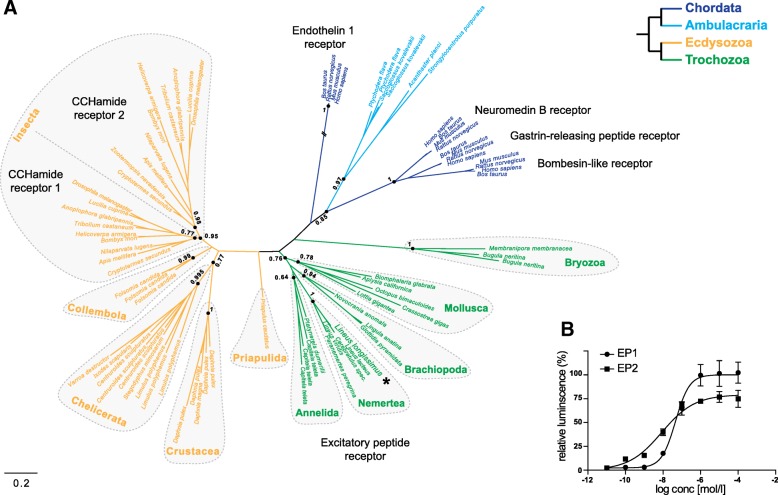
Analysis of EP/CCHamide receptors. **a** Phylogenetic analysis of protostome EP/CCHamide receptors and related deuterostome receptors. Color coding according to the phylogenetic group as depicted in the simplified tree on the upper right corner. SH-like support values are shown for the indicated nodes. Scale bar on the lower right corner shows amino acid substitution rate per site. The endothelin 1 receptor branch was shortened to half its size (indicated by the two crossing lines). Asterisk highlights the *L. longissimus* receptor that was biochemically characterized. **b** Activation of the *L. longissimus* EP receptor by the two *L. longissimus* excitatory peptides EP1 (EC_50_ = 78 nmol/l) and EP2 (EC_50_ = 59 nmol/l)

In the nemertean *L. longissimus* we identified two excitatory peptide transcripts that seem to be the result of alternative splicing*.* The peptides differ only in a few amino acids at the N-terminus of the predicted EP/CCHamide peptide, whereas the rest of the two sequences are identical in their amino acid and nucleotide sequence. PCRs using larval cDNA and two specific primer pairs that were designed in the region where the two transcripts vary confirmed the presence of two separate transcripts (Additional file 2). We only identified a single ortholog of EP/CCHamide receptors in the transcriptome of *L. longissimus*. A phylogenetic analysis shows the orthologous relationship of protostome EP and CCHamide receptors and the chordate neuromedin B/bombesin and endothelin related receptors (Fig. 2a). The analysis also shows the duplication of the CCHamide receptors in the early insect lineage, which is not present in Collembola, but in as distantly related pterygote insects as dipterans and termites. This receptor duplication within pterygote insects and its absence in Collembola is in accordance with the presence of two CCHamide paralogs in pterygote insects (Fig. 1b).

We cloned the *L. longissimus* EP receptor into an expression vector, expressed it in mammalian cells and tested its activation by the *L. longissimus* EP1 and EP2 peptides. Both peptides activated the receptor at similar, nano-molar concentrations with EC_50_ values (half maximal effective concentration) of 78 nmol/l for EP1 and 59 nmol/l for EP2 (Fig. 2b).

### EP is expressed in neurons that project from the apical organ to the ciliary bands of *L. longissimus* larvae

**Fig. 3:**
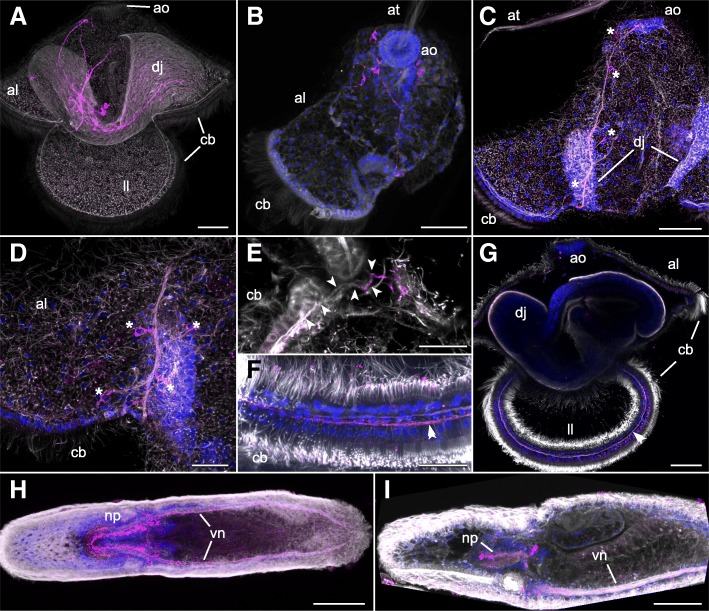
``Immunohistochemical analysis of EP in *L. longissimus* larvae and juveniles. **a** Side-view of a larva with advanced developing juvenile, showing nerve underneath the ciliary bands and the EP positive nerves in the apical lobe and in the juvenile. **b** Top-view of the apical part of a larva, with apical organ and ciliary band of the apical lobe, showing network of EP positive nerves. **c** Side-view of the apical part of a larva, showing EP positive nerve running from the apical organ towards the ciliary band. Asterisks indicate EP positive cell bodies. **d** Side-view of the EP positive nerve before the innervation of the ciliary band, including EP positive cell bodies of the nerve cells. Asterisks indicate EP positive cell bodies. **e** Innervation of the nerve underneath the ciliary band by a more proximal EP positive part. Arrows follow the nerve with strong proximal EP signal in the beginning. **f** Close-up of EP positive signal in the nerve underneath the ciliary of a lateral lappet after strong signal amplification. Double arrow indicates the same nerve as in (**g**). **g** Side-view of a larva with developing juvenile, showing EP positive signal in the nerve underneath the ciliary after strong signal amplification. Double arrow indicates the nerve. **h** Top-view of a juvenile with broad EP positive signal in the neuropil and the ventral nerve cords. **i** Side-view of a juvenile with broad EP positive signal in the neuropil and the ventral nerve cord. *Orientation*: all larval pictures are oriented with the anterior side of the developing juvenile to the left. *Abbreviations*: al = apical lobe, ao = apical organ, at = apical tuft, cb = ciliary band, dj = developing juvenile, ll = lateral lappet, np = neuropil, vn = ventral nerve cord. *Color coding*: gray/white = anti-tyrosinated tubulin staining (cilia and nerves), magenta = anti-EP staining (EP positive nerve cells), blue = DAPI staining (nuclei). Scale bars indicate length of 50 μm in A-C and G-I, and 20 μm in D-F

We visualized EP expressing neurons in *L. longissimus* pilidium larvae using polyclonal antibodies against the C-terminal residues CAAGNGamide. The larval staining revealed EP positive neurons in the apical part of the larvae as well as in developing juveniles inside the larvae (Fig. 3a). The larvae showed the apical EP positive nerves already in 5-day old young larvae before development of the juveniles. The EP positive nerves of the larvae project from the lateral organ towards the ciliary bands at the anterior end of the future juvenile (Fig. 3b, c). The antibodies stained axons as well as somata of a variable number of cells (Fig. 3c, d), possibly depending on the age of the larva. The projecting EP positive neurons directly innervate the prominent neurons underneath the ciliary band at the point where the ciliary bands of the apical lobes and lateral lappets meet (Fig. 3e). With strong excitation and sensitive detection setting, the nerves of the ciliary bands themselves also showed weak EP immunoreactivity (Fig. 3f, g), however, the intensity of the labelling was weaker than the one of the nerves that project from the apical organ and we could not detect EP positive cell bodies that might belong to the ciliary nerve. The EP positive neurons of the larvae and the developing juveniles are not connected. Hatched juveniles show prominent EP staining in the neuropil and in the two longitudinal ventral nerves along the whole body (Fig. 3h, i). We did not observe any EP positive signal in larvae or juveniles when the antibodies were preabsorbed with synthetic EP prior to incubation.

### EP influences the swimming behavior of *L. longissimus*

**Fig. 4:**
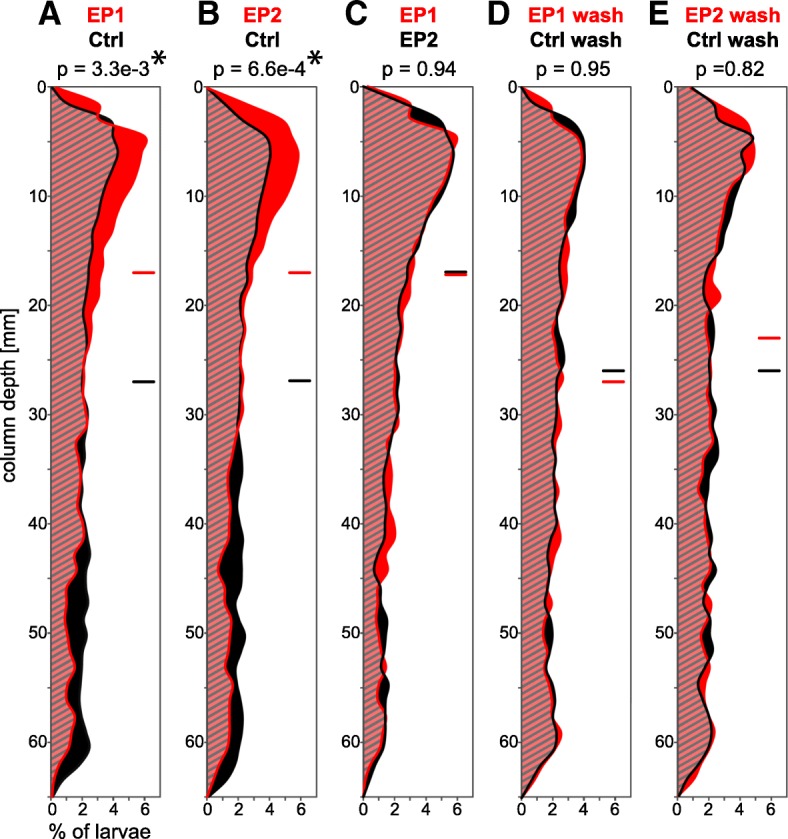
Effect of EP on the vertical distribution of *L. longissimus* larvae in a water column. Comparisons of **a** control and EP1 exposed specimens, **b** control and EP2 exposed specimens, **c** EP1 and EP2 exposed specimens, **d** control and EP1 exposed specimens after several water changes, **e** control and EP2 exposed specimens after several water changes. Y-axis shows water depth of column, X-axis shows the percentage of larvae. Red and black lines show distribution of larvae under the condition stated above the columns. Horizontal lines indicate average swimming height. *P* values compare the distribution of the two samples (two-sample Kolmogorov-Smirnov test). EP1 and EP2 concentration = 5 μml/l. Ctrl = control, wash = washout

We tested the influence of the predicted EP1 and EP2 peptides on *L. longissimus* specimens by soaking them with synthetic peptides. When we exposed 2–3 week old planktonic pilidium larvae to 5 μmol/l EP1 or EP2 and recorded their swimming behavior in a vertical column, their average swimming level was shifted upwards, and larvae concentrated in the upper part of the water column (*p* = 3.3e-3 for EP1 and 6.6e-4 for EP2) (Fig. 4a, b, Additional file 6: Video S1). The vertical distribution of larvae exposed to EP1 and EP2 seemed similar (*p* = 0.94) (Fig. 4c) and after washout of the peptides, the formerly treated larvae returned to a similar distribution as washed out control larvae (*p* = 0.95 for EP1-washout/Ctrl-washout and 0.82 for EP2-washout/Ctrl-washout) (Fig. 4d, e) (see also Additional file 3, Additional file 4). Juveniles did not show any obvious reaction at any tested concentration between 50 nmol/l and 50 μmol/l.

### EP causes an increase in the beat frequency of apical and lateral ciliary bands of *L. longissimus* larvae

**Fig. 5:**
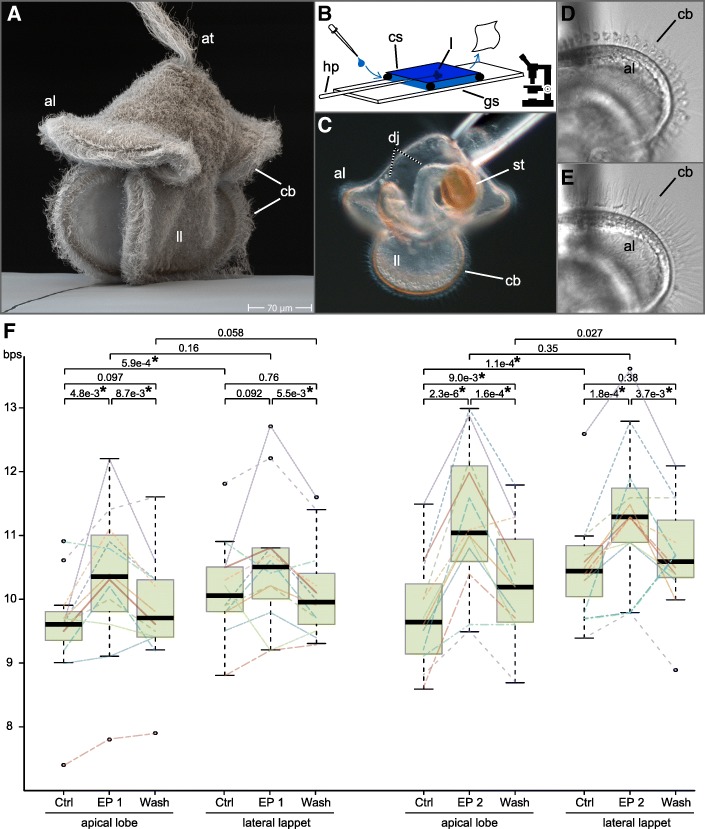
*L. longissimus* pilidium larvae and the influence of excitatory peptide on their ciliary beating. **a** SEM picture of a *L. longissimus* larva. **b** Experimental setup to record the ciliary beating and the influence of excitatory peptide on a microscope slide. **c** Light microscopic picture of *L. longissimus* larvae with a developing juvenile on a holding pipette. **d** Beating ciliary band of the apical lobe. **e** The same ciliary band as in D, after overexposure to 25 μmol/l EP2. **f** Boxplot of the ciliary beat frequency of the apical lobes and lateral lappets under normal conditions, after exposure to either 10 μmol/l EP1 or 5 μmol/l EP2, and after peptide washout. Bold horizontal line indicates median, box indicates upper and lower quartile, whiskers indicate variability outside the upper and lower quartile, circles indicate outliers. Lines within the graph indicate measurement points of individual larvae. *P* values were calculated with a paired t-test. Asterisks indicate significance with *p* < 0.02. al = apical lobe, at = apical tuft, bps beats per second, cb = ciliary bands, cs = cover slip, Ctrl = control, EP = excitatory peptide, gs = microscopic glas slide, hp. = holding pipette, dj = developing juvenile, l = larva, ll = lateral lappet, st = stomach

To test whether the upwards shift of the swimming distribution of *L. longissimus* larvae after exposure to EP may be due to a change in the beat frequency of their locomotory cilia (Fig. 5a), we recorded and quantified the beat frequency of locomotor cilia before, during and after exposure to excitatory peptide (Fig. 5b,c, Additional file 5, Additional file 7 Video S2). When larvae were exposed to concentrations of 25 μmol/l or higher of EP1 or EP2, the cilia stopped beating rhythmically and started standing up straight instead, while vibrating with a high frequency (Fig. 5d, e, Additional file 8: Video S3). At lower concentrations, we detected a significant increase of the ciliary beat frequency (cbf) of the outer ciliary bands of the lateral lappets and apical lobes (Fig. 5f). EP1 seemed to be less efficient than EP2, as 10 μmol/l EP1 were necessary to observe significant changes in the cbf, compared to only 5 μmol/l EP2. When the peptides were washed out, the cbf decreased again. We were not able to determine a possible effect of EP on ciliary arrests which occur in combination with muscular contractions of the lobes. Such contractions seemed to be inconsistent between individual larvae (possibly based on the experimental setup) and increased with the time the larvae were stuck to the holding pipette. However, it has to be mentioned that such contractions can be a mechano-sensory response in pilidium larvae that is related to feeding [77]. The ciliary bands of the lateral lappets showed under control conditions a significantly higher cbf than the ciliary bands of the apical lobes with an average of 10.34 beats per second (bps) and 9.66 bps, respectively (*p* = 1.17e-7, *n* = 24). Exposure to EP1 or EP2 cancelled this difference, so the cbf of the lateral and apical ciliary bands became more similar with 10.98 bps and 10.79 bps, respectively (*p* = 0.083, *n* = 24; see Fig. 5f for differences between EP1 and EP2 treated animals).

## Discussion

In our study we found a wider distribution of EP/CCHamide-type peptides in protostome clades, in addition to those described from arthropod, molluscs and annelid species: we found EP/CCHamide-type peptides in ecdysozoan clades like tardigrades, onychophorans, nematomorphs, loriciferans and priapulids, and in spiralians like rotifers, entoprocts, brachiopods and nemerteans. We show that a group of heteronemerteans possess an additional glycine residue at the C-terminus of the peptide, which is absent in other spiralian. Our peptide-receptor binding assay shows that this modification did not change the peptide-receptor pairing. Our immunohistochemical results show that the planktonic pilidium larvae of *L. longissimus* possess EP positive nerves that project from the apical organ to the ciliary bands. The exposure of the *L. longissimus* larvae to synthetic peptides of their endogenous EPs led to an upwards shift in their vertical distribution and closer observation revealed that it increases the beat frequency of the locomotory cilia of their ciliary bands, representing the first experimental observation of a behavioral effect of a neuropeptide in a nemertean.

### Evolution of EP/CCHamide-type neuropeptide signaling

In our sequence analysis of EP/CCHamide-type neuropeptides, we found several characteristics that are shared between spiralian and ecdysozoan EP/CCHamide-type neuropeptides. EP/CCHamide-type neuropeptides usually possess two cysteine residues, an amidated C-terminus and a propeptide structure where the signal peptide is directly followed by a single copy of the predicted ligand. A similar propeptide structure with a single N-terminal peptide as well as an amidated C-terminus of the mature peptide is also characteristic for the related deuterostome neuromedin B and gastrin-releasing peptides [3, 79]. Cysteine residues, however, are only present in endothelins, but those peptides possess four cysteines instead of two [80, 81]. The only true indication for an orthology of these deuterostome peptides with the protostome EP/CCHamides are therefore - as previously shown - their receptors [1–3]. Other common features of protostome EP/CCHamide-type peptides are the presence of either two consecutive glycine residues or a glycine and an alanine residue between the second cysteine and the amidated C-terminus, as well as the presence of an aromatic amino acid and often a histidine residue between the two conserved cysteine residues. The region between the two cysteine residues shows strong similarities between annelids, molluscs, and the nemertean sequences and to a certain extend also in brachiopods. The corresponding region of the entoproct and rotifer EPs, however, seems more similar to the arthropod EP/CCHamide-type neuropeptides. This could indicate that the trochozoan (Annelida, Mollusca, Nemertea, Brachiopoda) EPs might have diverged more from the ancestral protostome EP/CCHamide peptide. The additional C-terminal glycine residue that is present in certain heteronemerteans is an evolutionary novelty of this group. This heteronemertean modification, however, did not change the peptide-receptor specificity, as it still activates the *L. longissimus* EP/CCHamide-type receptor.

### Physiological role of the EP/CCHamide-type peptides in *L. longissimus* larvae

There are only few studies that test the behavioral effect of neuropeptides on trochozoan larvae [57–61] and those focus on peptides other than the EP/CCHamide and none of them includes nemerteans. We observed an increase in the ciliary beat frequency of the locomotory cilia when we exposed *L. longissimus* larvae to synthetic *L. longissimus* EPs and an upwards shift of the vertical distribution of these larvae in a water column. An influence of neuropeptides on the ciliary based swimming of trochozoan larvae has also been reported for other neuropeptides [57–60]. The EP positive nerves in *L. longissimus* larvae project from the apical organ to the ciliary band and seems to directly connect sensory input and locomotory output organ. The apical organ of pilidium larvae is presumed to have a mechano-sensory function, based on the presence of collar cells and the long, bundled apical tuft [82], but an additional function like chemosensation cannot be excluded. The influence of EP on ciliary locomotion of *L. longissimus* larvae and the broad expression in the prominent ventral nerve cords of the juveniles stands in contrast to the myo-excitatory effect of EPs in different adult/juvenile annelid [6–8, 10] and mollusc [15] species and the repeated association of EPs and CCHamides with feeding or the digestive system in annelids [6–10], molluscs [15, 16] and insects [5, 23, 36–42] (see also Table 2). A direct association of the EP positive cells with feeding behavior can likely be excluded, as at least the feeding reflex of the larvae is triggered by sensory cells that are situated directly along the ciliary band [77] and are therefore independent of the apical organ. However, we cannot exclude an indirect association with feeding, by for example influencing the foraging behavior based on other stimuli such as for example chemosensation. In the larvae of the annelid *Platynereis dumerilii*, it has been shown that the ciliary beating of larvae and their vertical distribution in a water column can be influenced by a variety of different neuropeptides [57] and their endogenous release is likely depending on different specific inputs. The same observed reaction – namely an increase in ciliary beat frequency – can therefore be the result of different underlying behaviors like for example phototaxis or chemosensation. Based on our experiments, it is therefore difficult to speculate about the underlying trigger that naturally causes a release of EP and a resulting change in locomotion in *L. longissimus* larvae. Also, an additional secondary effect that is unrelated to locomotion, but might for example modify the feeding reflex, cannot be excluded. However, it has been shown that a single type of neuropeptide can be involved in different behavioral aspects during the life history [58, 83] and ancient peptides can trigger larval specific behaviors [60]. It has also been demonstrated that the same peptide can have opposite effects on the same organ of closely related species [84]. Therefore, only a larger comparison of the effect of EPs on larvae from other trochozoans, including species with feeding as well as species with non-feeding larvae, could give more insight into the conservation of the here described effect of EP/CCHamide-type peptides in trochozoan larvae.

## Conclusions

Our results show that EP/CCHamide peptides are broadly conserved in protostomes. We show that the EP increases the ciliary beat frequency of *L. longissimus* larvae, which shifts their vertical distribution in a water column upwards. Endogenous EP may be released at the ciliary band from the projections of apical organ EP positive neurons to regulate ciliary beating. A locomotory function of EP in *L. longissimus* larvae, compared to the association of EP/CCHamides with the digestive system in other animals, suggests either an integration of EP into an indirect aspect of feeding like for example the regulation of foraging behavior, or an integration of orthologous neuropeptides into different functions.

## Additional files

Additional file 1: EP and CCHamide precursors sequences used for Fig. 1. (DOCX 21 kb)

Additional file 2: Primer and sequences of *L. longissimus* EP1, EP2 and EP-receptor. (DOCX 36 kb)

Additional file 3: Individual measurements for the vertical distribution shown in Fig. 4. (XLSX 112 kb)

Additional file 4: KS test results of the vertical distributions shown in Fig. 4. (TXT 10 kb)

Additional file 5: Table with ciliary beat frequency measurements of individual larvae used for Fig. 5. (XLSX 11 kb)

Additional file 6: Video S1. Time laps (4x) of the swimming behavior of EP exposed larvae and untreated larvae. (AVI 11407 kb)

Additional file 7: Video S2. Ciliary beating of apical and lateral ciliary bands of a single larva under control conditions, after exposure to 5 μM EP2 and after EP washout. (AVI 3056 kb)

Additional file 8: Video S3. Larva on a holding pipette under control conditions and after exposure to high concentrations of EP (50 μM). (AVI 12376 kb)

Additional file 9: Sequences used for the phylogenetic analysis of the EP receptors in Fig. 2. (FASTA 41 kb)

Additional file 10: Alignment used for the phylogenetic analysis of the EP receptors in Fig. 2. (FASTA 31 kb)

Additional file 11: Fasttree output file of the phylogenetic analysis of the EP receptors (Fig. 2). (TXT 7 kb)

## Data Availability

Sequence files, individual measurements and example videos are provided as Supplement. Raw data videos are available from the authors upon reasonable request. Genbank accession numbers for *L. longissimus* EP1, EP2 and EP receptor are: MN068403 - MN068405.

## Abbreviations

bps: beats per second
cbf: ciliary beat frequency
*D. melanogaster*: *Drosophila melanogaster*
EC_50_: Half maximal effective concentration
EP: Excitatory peptide
*L. longissimus*: *Lineus longissimus*
L11: Elevenin
