# Supplementary material for: A nemertean excitatory peptide/CCHamide regulates ciliary swimming in the larvae of Lineus longissimus
Source: Front Zool. 2019 Jul 10;16:28. doi: 10.1186/s12983-019-0326-9 (PMC6617912; doi:10.1186/s12983-019-0326-9)
Supplement: Supplementary file 1 — EP and CCHamide precursors sequences used for Fig. 1. (DOCX 21 kb) [file 12983_2019_326_MOESM1_ESM.docx]

**EP/CCHamide precursor sequences**

Signal peptide

Cysteine

Amidation and cleavage site

Cleavage site

**Predicted active peptide**

>Nemertea_Lineus_longissimus_01

MSTYGVWSLLVLVFIYLCLGSYTVAG**NSGTDGKCKGRWAIHACAAGNG**GKRSDPRLQIHIPSERQRTLQDMLEILRSRLLEDEANELEEEELPTYETTENDDMWNRLYSKLKERQSYAVAK

>Nemertea_Lineus_longissimus_02

MSTYGVWSLLVLVFIYLCLGSYTVAG**SKCKGRWAIHACAAGNG**GKRSDPRLQIHIPSERQRTLQDMLEILRSRLLEDEANELEEEELPTYETTENDDMWNRLYSKLKERQSYAVAK

>Nemertea_Lineus_ruber

MSTNGVWSLLVLAIIYVCIGSYTVAG**SKCKGRWAIHACAAGNG**GKRSDPRLQIHLPAERQRNLQDMLEILRTRLLEDEANALEEEELPTYETTENDDMWNRLYSKLKERQSFGVAK

>Nemertea_Baseodiscus_unicolor

MSSNGVWSLIVLAFIYLCLGSCTVSA**SKCKGRWAIHACGAGN**GKRSDPRLQIPDPSARRQKLEDMIELLRTRLLEEEAEEMTEELPTYETSDLTDDLWDKLYTKLKADQSYGRLQ

>Nemertea_Cerebratulus_spec

MSTYGVWSLLVLAFIYVCLGSYTVAG**SSSQDGKCKGRWAIHACAAGNG**GKRSDPRLQLNGPSERQRNLQDMLAILRSKLIEEEVNELEDEELPTYETTENDDMWNRLYSKLKESQSYADAK

>Nemertea_Lineus_lacteus

MSTYGVWSLLVLAFIYLCLGSYTVAG**SKCKGRWAIHACAAGNG**GKRSDPRLQIHLPLERQRNLQDMLEILRSRLLEDEANELAEEELPTYETTENDDMWNRLYSKLKERQSYGVAK

>Nemertea_Malacobdella_grossa

MLQRVIWTSAFILLFINFSLARR**AGKCRGRWAIHACAAGN**GKRSGPMYRPEDDLVELNNDRQKLVDLIQQLKSQLQTSPPEPAQIPLSSRFDVREEEPTIERSSELWKRLLSKIRSRDLLNA

>Nemertea_Nipponemertes_spec

MKTLASWSLILLATLFIYIASYHTANA**SKCKGRWAIHACAAGN**GKRSESPYPSDGFDHRPGAGRQTLEDMIQELRKRIGDKARFMTSDPRDAEGNNEQPQEADSSEIWRRLYMKMRARSVMSD

>Nemertea_Paranemertes_peregrina

MNLRVFWSSAFILLIVNFTSARA**SKCRGRWAIHACAAGN**GKRSGPLYPPDDDTDSDAAADNRQKLVDLIQQLKLQAQPQ

>Nemertea_Tubulanus_polymorphus

MRTWATWSVFLMAMVYLCLTFKTVSA**GKCQGEWFRHSCLAGNS**GKRSDSHIENRSKSEDYLKKLLSLLKTEIEMKSLPSSPVPEQEEADERPSRDYNTDKTISEPLQALWEKFLMRTREQEDMTRRK

>Brachiopoda_Lingula_anatina_XP_013414745.1

MRIRLDPWVVIPLAVVLACLLSHKTDA**NKCKGPWRVHGCFGGN**GKRTQPEPRPHHNELLAKQLNLAEIGILKKILKATRTERMDFLNSDKINSKSLSKHQHDRQAEYRTTRLPLLPLQSNSHLTPEKMFDGNLKDNHNRINLTEIYFQLKRTTLRRLFQMLQRKWNP

>Brachiopoda_Novocrania_anomala

MKSLDCWTILAVSIIYACFCTFVTEG**GKCRQWSSHHCWGGN**GKRGEQETDGSRDRIIKLIDKILQASKNRHIEDNAENIFERNTYQHKTQTESMEPRSLVHSRDQRQHMLRDWLLEVKNSRR

>Annelida_Dinophilus_gyrociliatus

MERLRLLWIFISVLGTLSFLAEG**GKCPVWALHACAGGN**GKRSEATNERESGLRQIINQLRSLQRENFEKPRKFTSDFPFFEKFLRKSEKGDSIWDEKRNVFRNF

>Annelida_Owenia_fusiformis

MYLGERLCSAFALWLLLNGCSLVTG**KCSGRWAIHACAGGN**GKRSQDNAIDYKVNTLNDDFYGRLLQKLMPQTSEENLPFQHVELKRNTLPDGPISLPYRGVLPMSESGSDPESYELSYDYNPKSRDALRGLLQKLDGRNKKH

>Annelida_Perinereis_vancaurica_BAB83127.1

MDRTGIWYLLLVSILYIWTGQKVSG**KCTGPWAIHACGGGN**GKRSDVEAGGLGDREKESRISLQRILRGFGEYSNEDEDSTHFGDPDLSNYVEDVLSQQPDLAYIQKENRKQQSLLRKLVAMLKLRQNLQNLQN

>Annelida_Eisenia_fetida_BAA09458.1

MRRPVTNIMLLVICYLWCHIQSTSG**GKCAGQWAIHACAGGN**GKRSDIDDLVRPTDLGTDEERPDIGLQEARGGERGEGGGGREGDGYRGDRDNDDLRSELSEGRQLLEDKQLLDIVNRRPRPPTPLESALGRLRSLLRTTDFDVDRREEEMKPAQWQEEETPRRKNTDKNSRYNIKDSLTMLRSILDDIEGRLDSQPVSISERKTRGYADDVENDSYSLQKRSGDQ

>Annelida_Platynereis_dumerilii_AHB62363.1 (extended N-terminus taken from Conzelmann et al. 2013, BMC Genomics)

MDRTGLWYLLLVSVLYIWTGQKVSG**KCSGQWAIHACAGGN**GKRSEGALSSIGDRERDSRMSLQRILRGFGEYSNEVEDQNRFASEPELSNYEDVLSQPQPDLTYLQPDLTYLQKENRKQKSVLRKLFTILKLRQNRQNLQN*

>Mollusca_Charonia_tritonis_AQS80510.1

MMKTDTAQTLCIALTSFVVLSAVFSPTEG**KCAGRWAIHACWGGN**GKRSDPSLNVAPSPSVLRQLLLRNRPVYAQALDSSEELPAVAPQPADDLGSSYNSDFSDYNVLPASPSVSRLTALLRTLRSLQKENEVLP

>Mollusca_Reishia_clavigera_BAQ25803.1

MKTDIQQALCLAMTLFVIVSTVIKPSEG**KCYGKWAMHACWGGN**GKRSDPSADLAPNPSVLRQLLLRNPPALDSREDASSYSDGLPEYNVPPPPAPSVSRLAALLRTLRTLQRENDALP

>Mollusca_Reishia_clavigera_BAQ25802.1

MTTRGSCVMCAAMMSLLILSSIVSNAEG**KCSGKWAIHACWGGN**GKRSGIPAEEDDNTAKSLDLLRRLLRRKVSSSPYLPSLEDTVYVPVQPEDFLSSSESLPSSLSSSSSLLSSSSPFEDGFSEDVEEAEAAAASFVPSKNRLASLTTILRKLQQGRDDFQ

>Mollusca_Crassostrea_gigas_XP_011422247.2

MRNPSSFVLFSATLCVYFTVQISSVSA**SKCKGPWANHMCFGGN**GKRSWSPPVQEPEMNRKDDELGRTMLRNVLLKRLNTYPSMSSYYSDSESFYPMGSDFTEEGDSMSRENELRQLLKEQILRKEMAALVGDDDVYE

>Entoprocta_Loxosoma_pectinaricola_Lpec.Gene.102227

MAIKKMSSVVILFVLLWSVTLIYETQG**IPSNCNLFGHRCFGG**GRKRSDLSSMKGIPLLEKLQQLVDKDTESDGFKQQTPSLLHLISRLSDYPPPEAERFQEKEEGPTFLQE

>Loricifera_Armorloricus_elegans_01_Aele.Gene.8385

MRSSMVGLLALVVVLGVIEQADLVRG**DCLSYGHACLGGL**GKRSRAAAEKPPEREAAVRGKAMENLILDLIANQRTRKWWPWERQPYRDLS

>Nematomorpha_Paragordius_varius_Pvar.Gene.19198

MNFGYRLLLWNVLAFQLVLLTIAAKNKCTMFGHSCLGGFGKRQDGDDGAANKIRQLMESPEDVFISNTDYYDANGDRVDPIVYFKDRKFSDQLANLRYKNRMNNFLLRKRSPRFDYRPE

>Onychophora_Peripatopsis_capensis_Pcap.Gene.69447

MLAHQSIMKCLMVLLCLCYMTIAVSG**LKGCANFGHSCLGGH**GKRSGETDTIKKQKLLASLESRNNNFSDEDGYPAFNIEASSEDPENHGIESDSRFSDENVHGIDRIASPVLRKWMALMQAARR

>Orthonectida_Intoshia_linei_Ilin.OAF70707.1

MHMNVLLIFLFVFFFERAIKG**VIIRDSDMDSYLNQKCQGIWRVHFCLSAN**GKRSYRFRTIPHKRNVNADYEDFSELTEFI

>Rotifera_Adineta_vaga_01_Avag.GSADVT00010911001

MARSSAHITPMYFTCFLIVILLLQFTQPIQS**LALSSLCGQFGHSCFGGNW**GKRELSTASIATDVLRSNFDDLDGEMATTSEDEEMLIKNLLLEEIRLSLLRQRVRHLLKLE

>Rotifera_Adineta_vaga_02_Avag.GSADVT00044675001_

MNSRSVFLVPFRFPIFLLIISSLLILQSSRPVQS**ISLSRLCGQFGHSCFGANW**GKRSNADATSTQFITFDLNDDDATRMPTVIQDDILEKLRMVKENKSTLIYHEYVFSCLYRNYIGNVGDNY

>Rotifera_Rotaria_tardigrada_01_Rtar.Gene.122222_and_Rtar.Gene.176799

MNSSSFIITRVRFSIGLLILWSLLFCQFPRPVQT**IALGQLCGRFGHSCYGGNW**GKRTLPNDLSEQYVLWNPNLNNEEIPVFPVIDRNSMSNNILEELQSELLRQRLRQLLELE

>Rotifera_Rotaria_tardigrada_03_Rtar.Gene.31633

MNSPSFIITHVRFPIGLLILSNLFFCQFPQPVQA**IALGQLCGRFGHSCYGGNW**GKRTLPNDLSEQYVLWNPNLNNEEIPVFPVIDRNSMSNNILEELQSELLRQRLRQLLELE

>Rotifera_Rotaria_tardigrada_04_Rtar.Gene.109356

MKRQSIVLAPVCFSYFLILILLFQSTQTVQA**IALSSLCGHFGHSCFGGNW**GKRELLASSLVPDIIQVNTDTNNDRIEIPDAYYKDLINDYFLEQLRSKLIREQLHQLLEQE

>Rotifera_Rotaria_tardigrada_05_Rtar.Gene.138730

MNRQSIVLAPVCFSYFLILILLFQSTQTVQA**IALSSLCGHFGHSCFGGNW**GKRELLASSLVPDIIQVNTDTNNDRIEIPDAYYKDLINDYFLEQLRSKLIREQLHQLLEQE

>Rotifera_Rotaria_tardigrada_06_Rtar.Gene.138731

MNRQSIVLAPVCFSYFLVIILLSQSTRPVQS**IALSSLCGHFGHSCFGGNW**GKRELLASSLAPDIIQESTDTNDDRTEIPTAYYKESMNDFLLEQLRSKLIRQRLRQLLELE

>Rotifera_Rotaria_tardigrada_07_Rtar.Gene.81709_and_Rtar.Gene.200251

MNSPSFIITHVRFPIGLLILSNLFFCQFPQPVQA**IALGQLCGRFGHSCYGGNW**GKRTLPDDLSQYILWNPNIKNDEKAVLPVFDRNSMSNYILEELRSELFRQRLRQLLDLE

>Priapulida_Priapulus_caudataus_01_Pcau.rna.tri.28822.1

MRTIEAVELCLIVTILACCLTATHG**GCHAYGHACLGGM**GKRADDAALQSSLQLSRDDTQRPRTAHGYYNQLRQLLQRVAALDRQKQTARMTSQNDDFRRDVGDYSDRFYPVDERRTYADDDGGIATGDLGKLIDQTRRLGGISKKWQQPLMDPSSMGDDSEDVEGINDVRWLRKRSISTSRRR

>Priapulida_Priapulus_caudataus_02_Pcau.rna.tri.49310.1

MCYTTGWLHFASVLVVSCCFIAQASG**SCAAYGQSCLGGF**GKRTDLAESTADLGLGLPYADEPQTSESLAVLLDKLRELAAGRGAQGETRQSLEQAYEQPQLLRRIINHMYENSHRVRRRRR

>Priapulida_Priapulus_caudataus_03_Pcau.rna.tri.28343.1

MDTSFTPTVLVLLFLFARVEG**GCWSYGHSCFGGF**GKRSETSKNTKMTPTVANTQHDVVNRINEMQRLLEGLSKLSESTRFRRSDLPQR

>Priapulida_Halicryptus_spinulosus_01_Hspi_Locus_60750.0_Transcript_4/0

MKSSDVMKISFLFVMIGCCLG**TTKGGCLSYGHACLGGM**GKRSEETLQQANTYNDNILQNNDRSNELFAQFKRLLLRAAKLNDESIDELLPMQQAYGDYPDELTLGSSYSYTPAGNTGQLNDDMESRFEEIVDKLHSSDIRRNAAGKTRFRNHMERYDRK

>Priapulida_Halicryptus_spinulosus_02_Hspi_Locus_62381.0_Transcript_2/0

MQTTDAILCVLLCSLSVIATVHG**GCLRYGHACLGGF**GKRSDMPEIKEENREMINDNLNDLTADEVKQFVRRAAYELEKARYMQYGGERTGGQAYYDNTGAGDDSRANAVGGSKSDLYKYLLAAATAAHQKRKGNEPIWNRRNEALYNLDNSQ

>Priapulida_Halicryptus_spinulosus_03_Hspi_Locus_63478.0_Transcript_1/0

MYCCLHFICIFVVTCCFIAQASG**SCEAYGQSCLGGF**GKRAELSDNVGLQDLKLPYPNKEFMENDGSSLKNFLEKLQYFAEKRKPVAEERPLPEAAYKDPLLIKRLLAHIYENSHRVRRR

>Tardigrada_Milnesium_tardigradum_01_Mtar.Gene.19551

MSIATYALCVLTIGLSLILCVQSLKVNAGQGGNFRQQRRLK**GCALWGQSCLGGHF**KKRSQEEWQQNSDASNQQVNPLDERTLEVEAINDYDNLSLREKLDRLYALIEQQNYNTGNGYPYSADTQYMNKRTLYIPSGVPTWLKMARVG

>Tardigrada_Milnesium_tardigradum_02_Mtar.Gene.45401

MMYGYSNSDTIRFSCICIFITIICVQSLKVNAGQGGNFRQQRRLK**GCALWGQSCLGGHF**KKRSQEEWQQNSDASNQQVNPLDERTLEVEVINDYDSLSFERETRQTLCTDRTAKLQHRQRLPLLCVDTTA

>Tardigrada_Milnesium_tardigradum_03_Mtar.Gene.6654

MMYGYSNSDTIRFSCICIFITIICVQSLKVNAGQGGNFRQQRRLK**GCALWGQSCLGGHF**KKRSQEEWQQNSDASNQQVNPLDERTLEVEAINDYDSLSLREKLDRLYALIEQQNYNTGNGYPYSADTQYMNKRTLYIPSGVPTWLKMARVG

>Arthropoda_Daphnia_pulex_EFX80320.1_CCHamide-like_precursor,_partial

MHIFFYVIHVTAMLAIVSG**NCNKYGNACFGAH**GKRSDFKRTSAVDLSDQIWPVAANWNPTRPDEPIQERRQMKPLPALQLESVLVYNDIPRSAEHSRYLNQEDYNN

>XP_021952472.1 neuropeptide CCHamide-2-like [Folsomia candida]

MSTMTNKVSNNASSTCRLVGMICLVALVLLWGEVREAQA**GCQRFGHSCFGAH**GKRGGDVGATGGQQNGAELSPGESVYPAGPGAEYYPAGGFAPRVGMSPYLMDWLLSAHRGQPLSANYARQLQDDLNMADQRRK

>ANT96533.1 CCHamide 2 preprohormone variant A [Drosophila melanogaster]

MKSTISLLLVVICTVVLAAQQSQAKK**GCQAYGHVCYGGH**GKRSLSPGSGSGTGMGGGMGEAASGGQEPDYVRPNGLLPMMAPNEQVPLEGDFNDYPARQVLYKIMKSWFNRPRRPASRLGELDYPLANSAELNGVN

>NP_001097784.1 CCHamide-1 [Drosophila melanogaster]

MWYSKCSWTLVVLVALFALVTG**SCLEYGHSCWGAH**GKRSGGKAVIDAKQHPLPNSYGLDSVVEQLYNNNNNNQNNQDDDNNDDDSNRNTNANSANNIPLAAPAIISRRESEDRRIGGLKWAQLMRQHRYQLRQLQDQQQQGRGRGGQGQYDAAAESWRKLQQALQAQIDADNENYSGYELTK

>XP_023720028.1 uncharacterized protein LOC111871277 isoform X2 [Cryptotermes secundus]

MALQRCPSLLIVLTAIIVLMVEIDQSSAKR**GCSSFGHSCFGGH**GKRADEGVLLLPGADSDQQQRLMFPARETGSEDGEDTMIQPEGYGALSPASSASLVPSANRLSPFLRQWLQSYRRSTGDIEVE

>XP_023720024.1 uncharacterized protein LOC111871276 isoform X1 [Cryptotermes secundus]

MSCLSAPTRASVLGVAARVTVLLLVFGLAECAA**GSCLSYGHSCWGAH**GKRSGNVPATEEVPEDGAEAMAAIAAPEDTRWFLSKLVRRAEPSGRSKMWQRFGGLSLPSVGDRRHKETQWKNSPSEEDPDNAGESETVVRSRTVESSEDGPSGILVPASGEYPVQDSQDGEVVLMADEQAMRRMPQNLRVYKIMRPDRLRGPPSSYPMGIGGSSPEGKASGT
