# Supplementary material for: A nemertean excitatory peptide/CCHamide regulates ciliary swimming in the larvae of Lineus longissimus
Source: Front Zool. 2019 Jul 10;16:28. doi: 10.1186/s12983-019-0326-9 (PMC6617912; doi:10.1186/s12983-019-0326-9)
Supplement: Supplementary file 2 — Primer and sequences of L. longissimus EP1, EP2 and EP-receptor. (DOCX 36 kb) [file 12983_2019_326_MOESM2_ESM.docx]

**PCR Primer for isoform specific *L. longissimus* excitatory peptides.**

|  | Forward Primer | Reverse Primer (inside 3’ UTR) | length |
| --- | --- | --- | --- |
| EP1 (long) | GGTAATTCTGGCACGGAT | CCTTTACTCCACACAAACTC | 813 bp |
| EP2 (short) | CAGTTGCCGGTAGTAAAT | CCCTTTATACCTCTTTTCTTCC | 956 bp |

Annealing temp: 53°C

**PCR EP1 & EP2:**


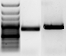


Lanes: Ladder (left), EP1 (middle), EP2 (right)

**Nucleotide sequence of *L. longissimus* EP precursors:**

(Additional stretch in yellow, flanking site of the additional stretch in green, primer site in bold, open reading frame underlined)

>Llongissimus_EP1

CACGTACCGTGTACAAAAAATCAATTATTAATCTCGCAGAAAGTGAATGAATGACGTACCTGAGATACAGGCTGCAACGCCGCCTGTCGGGGATCGTCCGCGATTTGGAGCGGAACATTCAGGGTTTGTCTTCTCAGATGCATACGGGGGTGGATTGAGTCATCATTTACGTTTAACTCTAGAGAGTCTTATATGAAAAGGGCGATTTTGATATTAACTTGGTTTATTTCACGGAAGGCGCGGTGCTGGTGATAAGTATACAATTGCGGCATGAGTACCTACGGAGTATGGTCTTTACTCGTTTTGGTGTTTATATACCTTTGTCTTGGAAGTTACACAGTTGCC**GGTAATTCTGGCACGGAT**GGTAAATGCAAAGGACGATGGGCAATACACGCATGCGCGGCAGGGAATGGCGGCAAAAGATCTGACCCTAGATTACAAATACACATACCATCGGAACGACAACGGACACTCCAGGACATGCTAGAAATACTCAGATCGAGGCTTCTTGAGGACGAGGCTAACGAATTAGAAGAAGAGGAACTACCAACATACGAAACGACGGAAAATGACGACATGTGGAATAGATTATACTCAAAACTGAAGGAACGCCAGTCCTACGCGGTCGCCAAATAATAAAACTTGGCGTCCGAGGTGGGCCACGTGTCGGACAACATATCAACCTGGACCACAACAATCCCTGTACAGAAGATTCTGCACCTAAAACGTGTCGGATATATTTATTTTTGTGACTAGCGCTGTAGTACCTATTGTGAGGCATCCAAATGAAGGAAATCCGTCAGTCTATCCGTTAAGCTAGATTAATACTTCAGAAATATTCGGAGTCACGACCAGCGCAACCAGGCCCAAGAAATGTCTCTCTCGTTTGCACCAATGGCGGATTTTATCGCTAAAATTCGAGAAGGCACTAATATACGAGCCTCGTGATATGGAGTACTAACCTTCAGTCTATCGGAGATGCAAGCAGTCTCATATGCGGTTATATTTTGTGTCAGCAACTCGTCCAATTGTTGTAATCAAAATGGCCTTCACCCCTTACAGACGTCATCGGAAGCAGTGTACACATTTACTGCTGACGTCATCATTGCAAGCACACTATGACACGAAGTATGCAAAA**GAGTTTGTGTGGAGTAAAGG**CAATAAGGTTACATGATGAAAACGTCTAGATTAGCCTGACCTTAGCTCCTTATACACATACGTCATCAGCAGTTTACACAAGAACTGCTGAGGTCATCAAGATAAATGTGCACTCTACGAAATGGAGCGGAAGAAAAGAGGTATAAAGGGCAATGG…

>Llongissimus_EP2

CACGTACCGTGTACAAAAAATCAATTATTAATCTCGCAGAAAGTGAATGAATGACGTACCTGAGATACAGGCTGCAACGCCGCCTGTCGGGGATCGTCCGCGATTTGGAGCGGAACATTCAGGGTTTGTCTTCTCAGATGCATACGGGGGTGGATTGAGTCATCATTTACGTTTAACTCTAGAGAGTCTTATATGAAAAGGGCGATTTTGATATTAACTTGGTTTATTTCACGGAAGGCGCGGTGCTGGTGATAAGTATACAATTGCGGCATGAGTACCTACGGAGTATGGTCTTTACTCGTTTTGGTGTTTATATACCTTTGTCTTGGAAGTTACA**CAGTTGCCGGTAGTAAAT**GCAAAGGACGATGGGCAATACACGCATGCGCGGCAGGGAATGGCGGCAAAAGATCTGACCCTAGATTACAAATACACATACCATCGGAACGACAACGGACACTCCAGGACATGCTAGAAATACTCAGATCGAGGCTTCTTGAGGACGAGGCTAACGAATTAGAAGAAGAGGAACTACCAACATACGAAACGACGGAAAATGACGACATGTGGAATAGATTATACTCAAAACTGAAGGAACGCCAGTCCTACGCGGTCGCCAAATAATAAAACTTGGCGTCCGAGGTGGGCCACGTGTCGGACAACATATCAACCTGGACCACAACAATCCCTGTACAGAAGATTCTGCACCTAAAACGTGTCGGATATATTTATTTTTGTGACTAGCGCTGTAGTACCTATTGTGAGGCATCCAAATGAAGGAAATCCGTCAGTCTATCCGTTAAGCTAGATTAATACTTCAGAAATATTCGGAGTCACGACCAGCGCAACCAGGCCCAAGAAATGTCTCTCTCGTTTGCACCAATGGCGGATTTTATCGCTAAAATTCGAGAAGGCACTAATATACGAGCCTCGTGATATGGAGTACTAACCTTCAGTCTATCGGAGATGCAAGCAGTCTCATATGCGGTTATATTTTGTGTCAGCAACTCGTCCAATTGTTGTAATCAAAATGGCCTTCACCCCTTACAGACGTCATCGGAAGCAGTGTACACATTTACTGCTGACGTCATCATTGCAAGCACACTATGACACGAAGTATGCAAAAGAGTTTGTGTGGAGTAAAGGCAATAAGGTTACATGATGAAAACGTCTAGATTAGCCTGACCTTAGCTCCTTATACACATACGTCATCAGCAGTTTACACAAGAACTGCTGAGGTCATCAAGATAAATGTGCACTCTACGAAATGGAGC**GGAAGAAAAGAGGTATAAAGGG**CAATGG…

**PCR primers for Full ORF *Lineus longissimus* EP receptor sequence with restriction site adaptors.**

| Forward Primer | Reverse Primer | length |
| --- | --- | --- |
| ACAATAGAATTCCGCCACCATGACGGACACAGACGACG (complete)  *ACAATA (5’ extension)*  *GAATTC (EcoR1 restriction site)*  *CGCCACC (extended Kozak sequence)*  *ATGACGGACACAGACGACG (GSP)* | ACAATAGCGGCCGCATGTCTACAGCGACTGTGAGTTG (complete)  *ACAATA (5’ extension)*  *GCGGCCGC (Not1 restrictions site)*  *ATGTCTACAGCGACTGTGAGTTG (GSP)* | 1180 bp |

Annealing temp: 63°C

GSP = gene specific primer

**Nucleotide sequence of *L. longissimus* EP receptor:**

(Primer site in bold, open reading frame underlined)

>Llongissimus_EP_receptor

GAATCAAAATGATACACCAGCGAAGACTCTAAGTTGAATCTCCTCATTCCGACGACAAAGAAGTCGAG**ATGACGGACACAGACGACG**GGGTCGTCGAGTACCAGTCGAACACGACCCGGGGAAACTCAACCTTCCTGTTAGGGAACGACACCACGAATGACACCGGGACTGGCGAGCTGATATACATCACGTCCACCCTTAACACTTCTACGGAAGCTTTAGTGGTGCCCATTGTCTTTGGGTTGATCTTCATCGTGGGAGTGATAGGAAATGGAACAACCATTTTCACAGTTCTAAAAAATAAATCGATGCGAAATGTCCCGAATGTTTACATTGTTTCGTTAGCGTGTGGTGATCTGTTGTTAATATTGATATCAGTACCTTGTATGGCTACGTTGTATACATTCATAGGATGGCCTTACGGGGCCGTCATGTGCAAAGTCACACATTTTCTTCGGACCATGTCATTAGGTGTGTCCGTGTTTACTCTGACCGCTCTTGGTGGCGACCGGTACACCGCTATCGTGAACCCTATGAGCAAGCACATGGGCAACCCGATTGCTCGGACGATCATAACCTCTGTATCTATATGGGTGGCTTCAATAGCTTTGGCAATTGTGGACGGGATCAGCGCCAGGATAAGCTACCATCAACATCGCGATTCCCCAGAAGTGTTTTACACTTGTCAAGAATACCCTGTGGACTGGGGCGATTGGTACCCAAAGTTTCATACGATATTTCGCTTCATCATCTATTTTGCACTACCAGTATACATCATCGCCTTGTTCTACGTCATGATCGCGCGTATTCTCGTCCACAGTTCGTATCACATGCCGGTAGAGGGCGGGTTGAAGAGTAGCCAAGGTAACAAACAGGTGGAGGCTAGGAAGAAGGTGGCCATTGTTGTCTTACTGTTGGTGGTCATATTCATTATCTGTTGGTTACCGAGGCACATTTTCAGTATGTGGTGGCACTTCGACCCGAATGATTACAACGAGTTTTGGCATATTTTTAAAGTGACTGGTTTCTGCTTGTGCTTTATCAATTCATGCGTCAATCCCATTACGCTGTATTTCCTTAGCAAGCAGTTTAGAAAGTATTTTAATCGTTATCTGTTCTGCCTGTGCAATAGGAAGAAGCGACTGCGAGCAGAGGTCACGTCTACGTCAATGTATAACTTTAACAGTACAGTCAGACGAACGAGTACCACCATGACAATGCTACC**CAACTCACAGTCGCTGTAGACAT**GGCGTGAGATTAAAACTTAATAGTATTGAACAAGTACGGGGATATGTCAGTTTAAGGAGTGAGCCGGAGAAGCCACACGCTTTCTGGGACAACATGGCGAATTACAGCGAAGGAGTCTTTGAAATACACCAGCTCTTACTACTGGCTGACACTTCCAAATTAGTACTGAGGAAAGTATTATTGCTGGTGTTGGAAATGAAAACCAGATTACGGGTGATTGCTTCAAAAAAGCCCTTTGAATGTGACATGCAAGGTCCGCCTTTAGCAGCCCTTTGAGAAAAACAGGTGAGGGAAGTCATCTAAAAGCAT
